# Attenuation of WNT signaling by DKK-1 and -2 regulates BMP2-induced osteoblast differentiation and expression of OPG, RANKL and M-CSF

**DOI:** 10.1186/1476-4598-6-71

**Published:** 2007-10-30

**Authors:** Ken-ichi Fujita, Siegfried Janz

**Affiliations:** 1 Laboratory of Genetics, Center for Cancer Research, National Cancer Institute, National Institutes of Health, Bethesda, Maryland, USA; 2 Department of Pathology, Roy J. and Lucille A. Carver College of Medicine, University of Iowa, Iowa City, Iowa, USA

## Abstract

**Background:**

Enhanced osteoblast-dependent osteoclastogenesis due to inhibition of Wnt/β-catenin signaling in bone morphogenic protein (BMP)-driven osteoprogenitors has been repeatedly implicated in the natural history of cancer-associated osteolytic lesions, but the mechanism of this bone loss is poorly understood.

**Methods:**

We examined the impact of secreted Wnt inhibitors from the Dickkopf (Dkk) family on pluripotent mesenchymal cells undergoing BMP2-induced osteoblastic differentiation.

**Results:**

We found that Dkk1 and -2 restored the Wnt3a-dependent reduction of alkaline phosphatase (ALP), Osterix and p53, indicating that mitigated Wnt/β-catenin signaling promotes certain aspects of early osteoblastogenesis through the BMP-p53-Osterix-ALP axis. Dkk1 and -2 increased the expression of the osteoclast differentiation factors, receptor activator of NF-κB ligand (RANKL) and macrophage-colony stimulating factor (M-CSF), upon stimulation with Wnt3a/1,25-dihydroxyvitamine D_3 _and Wnt3a/BMP2, respectively. The decoy receptor of RANKL, osteoprotegerin (OPG), was down regulated under the latter conditions. These findings indicated that Dkk1 and -2 facilitate osteoclastogenesis by enhancing RANKL/RANK and M-CSF/c-Fms interactions. Dkk4 weakly shared activities of Dkk-1 and -2, whereas Dkk3 was ineffective.

**Conclusion:**

Our results suggest that inhibited Wnt/β-catenin signaling in BMP2-induced osteoprogenitors *in vivo *promotes, on balance, the heightened formation of osteoclasts. Focally increased Dkk1 production by tumor cells in the bone may thus lead to focal bone loss.

## Background

The development of bone-resorbing osteoclasts is strictly dependent upon bone-forming osteoblasts and the balanced activity of both cell types is crucial for skeletal homeostasis [1,2]. Excess osteoclastic activity leading to focal bone loss is a common feature of human cancer, notably multiple myeloma (MM) [3]. Two factors supplied by osteoblast lineage cells are of critical importance for osteoclastogenesis: macrophage-colony stimulating factor (M-CSF)^1 ^and receptor activator of NF-κB ligand (RANKL) [2]. M-CSF is required for proliferation and survival of osteoclast precursors, while RANKL is critical for precursor differentiation into mature, multinucleated osteoclasts. In addition to these positive regulators, osteoblast lineage cells produce the major inhibitor of osteoclast differentiation, osteoprotegerin (OPG) [2]. Upon secretion into the extracellular milieu, OPG acts as a decoy receptor for RANKL, blocking the various signaling cascades activated by binding of RANKL to its receptor on pre-osteoclasts, RANK [4-6]. Altered expression of M-CSF, RANKL and OPG in osteoprogenitors may facilitate cancer-associated osteolytic disease by promoting osteoblast-dependent osteoclastogenesis.

Homeostatic bone remodeling requires coordinated integration of biological signals from numerous cellular signal transduction pathways including Wnt/β-catenin signaling [7]. Wnt/β-catenin promotes new bone formation by functioning as a positive regulator of osteoblasts [8-10] and, by up-regulating OPG [11-13] and down-regulating RANKL [12,14] on osteoprogenitors, negative regulator of osteoblast-dependent osteoclastogenesis. Wnt/β-catenin signaling in the osteoblast lineage is activated by binding of canonical Wnt ligands, such as Wnt3a, to a membrane-bound receptor complex that consists of Frizzled and low-density-lipoprotein receptor-related protein 5/6 (LRP5/6). Canonical Wnt ligands inhibit the degradation of β-catenin in the cytoplasm, leading to translocation of β-catenin to the cell nucleus where it cooperates with transcription factors of the T-cell factor/lymphoid enhancer factor family in regulating target gene expression [15,16]. In human beings, diminished and elevated Wnt/β-catenin signaling due to loss-of-function and gain-of-function mutations in LRP5 lead to osteoporosis [17] and osteopetrosis [18,19], respectively. Both phenotypes were accurately recapitulated in transgenic mouse models of LRP function [20,21], providing further evidence of Wnt/β-catenin's anabolic role in the bone.

Abnormal levels of secreted antagonists of Wnt signaling have been shown to shift bone remodeling in both directions. One class of Wnt inhibitors that blocks Wnt ligand binding to Frizzled includes secreted frizzled-related proteins (sFrps), which block both the canonical (β-catenin dependent) and the various non-canonical (β-catenin independent) Wnt pathways [22]. Mice deficient in sFrp1 exhibit high bone mass [23], whereas over-expression of sFrp2 has been implicated in the development of osteolytic lesions in MM [24]. Another class of Wnt inhibitors including Dickkopf (Dkk) proteins bind to LRP5/6 and Kremen1/2. This leads to suppression of canonical Wnt signaling but spares the non-canonical pathways [22]. Mice containing one wild type and one null allele of *Dkk1 *exhibit increased bone mass [25], whereas over-expression of DKK1 has been associated with osteolytic metastatic bone disease in prostate carcinoma [26] and MM [27]. These findings illustrate the significance of secreted Wnt inhibitors to bone health.

Secreted bone morphogenic proteins (BMPs), such as BMP2, are members of the transforming growth factor β (TGF-β) superfamily that can induce new bone formation *in vivo *[28]. BMP2 signaling begins with binding to serine/threonine kinase receptors on the cell surface, continues with phosphorylation of so-called restricted Smads forming complexes with common Smad in the cytoplasm, and culminates in the transcriptional activation of specific target genes in the nucleus [29]. Among the BMP2-induced genes important for osteoblast development is the zinc finger transcription factor, Osterix [30]. Upstream of Osterix is the transcription factor Cbfa1, a crucial determinant of the commitment of mesenchymal stem cells to undergo osteoprogenitor differentiation [31]. Farther upstream in this pathway is the cell cycle checkpoint protein and tumor suppressor, p53 [32]. Osteoblasts deficient in p53 exhibit enhanced ability to promote osteoblast-dependent osteoclastogenesis [32]. Noggin prevents BMP2 receptor binding [29], providing a tool to interrupt the BMP-p53-Cbfa1-Osterix axis in the osteoblast lineage.

Cross talk of Wnt/β-catenin and BMP signaling is known to play a role in the regulation of osteoblast differentiation and function, but the molecular mechanism by which Wnt/β-catenin influences BMP-induced osteoblastogenesis and, by inference, osteoblast-dependent osteoclastogenesis, is poorly defined. Here we used mesenchymal stem cells undergoing differentiation into osteoprogenitors *in vitro *to evaluate the mechanism by which Wnt3a/β-catenin modulates BMP2-driven pre-osteoblasts. Taking advantage of Wnt inhibitors from the Dickkopf family and the BMP inhibitor, Noggin, we demonstrated that inhibition of Wnt3a/β-catenin by Dkk-1 and -2 promotes certain aspects of osteoblast development through the BMP2-p53-Osterix axis. The concomitant up-regulation of M-CSF/RANKL and down-regulation of OPG indicated, however, that Dkk-1 and -2 also have the potential to promote osteoclastogenesis. The latter view is in line with a large body of clinical evidence on neoplasms constitutively expressing Dkk1 (MM, prostate carcinoma), showing that, on balance, this canonical Wnt inhibitor facilitates bone loss (osteolysis) *in vivo*.

## Results

### Dkk1 and -2 produced by mouse plasmacytoma cells reduce β-catenin in L indicator cells

**Figure 1:**
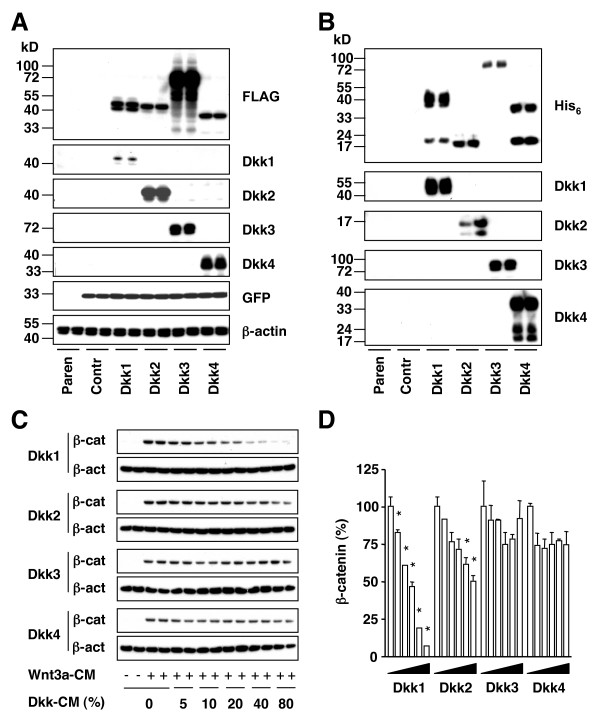
**Dkk1 and -2 secreted by MOPC315.4 cells attenuate Wnt3a/β-catenin signaling in L cells**. *A*, Constitutive expression of Dkk1-4 in stable transfectants of mouse plasmacytoma MOPC315.4. Total cell lysate (10 μg) was analyzed by Western blotting using Ab to FLAG, Dkk1-4 or GFP as indicated. β-actin levels (bottom) were determined as loading control. Untreated parental cells and cells transfected with GFP only (control) were included (bottom left). Protein size markers are indicated in kilodalton (kDa) to the left. *B*, Secretion of Dkk1-4 into the cell culture supernatant. MOPC315.4 transfectants (1 × 10^4 ^cells/cm^2^) were cultured for 3 days and 10 μl CM were analyzed by Western blotting using Ab to His_6 _and Dkk1-4 as indicated. Note that Dkk2 was secreted as an apparent cleavage product (<17kDa) of the intracellular protein shown in panel A (~40 kDa). *C*, Dkk1 and -2 inhibit Wnt3a-induced stabilization of β-catenin in L cells. Dkk-CM was added to the cell culture medium at final concentrations of 5, 10, 20, 40 or 80 percent (v/v) as indicated at the bottom. Zero Dkk-CM designates samples supplemented with 20% M-CM. Three hours later, Wnt3a-CM (indicated by plus symbols in the 2^nd ^line from the bottom) or L-CM (no Wnt3a; minus symbols) was added at a final concentration of 10%. Cells were cultured for 3 h and β-catenin (β-cat) and β-actin (β-act) were determined by Western analysis using 10 μg total protein per sample. *D*, Relative expression levels of β-catenin in L cells exposed to Wnt3a and Dkk1-4. Three immunoblots, including the one presented in panel C, were evaluated by densitometry and the ratio of β-catenin to β-actin was determined. This ratio was defined as one (100%) in cells stimulated with Wnt3a but not exposed to Dkk. Asterisks indicate significant drops in β-catenin in Dkk-treated compared to untreated cells (*p *< 0.05).

To evaluate the role of mouse Dkk proteins in cancer-associated focal bone lesions in mice, we generated four different sublines of mouse plasmacytoma MOPC315.4 that stably expressed Dkk1, -2, -3 or -4. Tumor cells were transfected with retrovirus that contained a bi-cistronic expression vector encoding one of the Dkk proteins in conjunction with the reporter, enhanced GFP. The reporter was used for enriching GFP-expressing cells by flow sorting, which increased, in the case of Dkk1, the fraction of GFP^+ ^cells from 70% to 90%. Similar enrichments were achieved with Dkk2, -3 and -4. All four Dkk proteins were readily detected by Western blotting of lysates of GFP^+ ^tumor cells using either Ab to the FLAG epitope (Fig. 1A top) or specific Ab to Dkk1, -2, -3 or -4 (Fig. 1A center). Western blotting with Ab to β-actin and GFP showed that parental MOPC315.4 cells or cells transfected with retrovirus expressing only GFP did not express any detectable Dkk protein (Fig. 1A bottom). Real-time PCR of Dkk1, -2, -3 and -4 mRNA levels confirmed the negative immunoblotting data presented in Figure 1A (not shown).

Western analysis of cell culture supernatants from the above-described MOPC315.4 transfectants demonstrated that all four Dkk proteins were secreted into the extracellular milieu (Fig. 1B). All four Dkk's were readily detected by antibody to the His_6 _tag (Fig. 1B top), FLAG (results not shown) or specific epitopes on the Dkk1, -2, -3 or -4 proteins (Fig. 1B bottom). MOPC315.4 cells left untreated or transfected with retrovirus expressing only GFP did not secrete Dickkopf protein (Fig. 1B, lanes labeled Parental and Control, respectively). Just like human DKK secreted by transient 293T human embryonic kidney cell transfectants [33], the secreted mouse Dkk proteins appeared to undergo post-translational modification including proteolytic cleavage and glycosylation, which produced distinct, highly reproducible bands on the immunoblots (Fig. 1B and data not shown).

To investigate whether the secreted Dkk proteins functioned as inhibitors of Wnt/β-catenin signaling, we performed the β-catenin stabilization assay in L cells [34,35]. Stimulation of L cells with Wnt3a-CM (obtained from Wnt3a-transfected L cells) resulted in a marked increase of β-catenin compared to L cells stimulated with L-CM (obtained from mock-transfected L cells), as shown in Figure 1C (compare lanes 3–4 with lanes 1–2). The Wnt3a-dependent accumulation of β-catenin was reduced in cells treated with Dkk1- or Dkk2-CM (obtained from transfected MOPC315.4 cells) in a dose-dependent manner (Fig. 1C and 1D). Dkk3-CM was ineffective, as expected, whereas Dkk4-CM, which was marginally effective in some experiments described below, was also ineffective in these experiments (Fig. 1D). These results clearly demonstrated that Dkk1 and -2 secreted by mouse plasmacytoma cells functioned as canonical Wnt/β-catenin inhibitor.

### Dkk1 and -2 inhibit Wnt3a-induced expression of ALP and osteocalcin (OCN)

**Figure 2:**
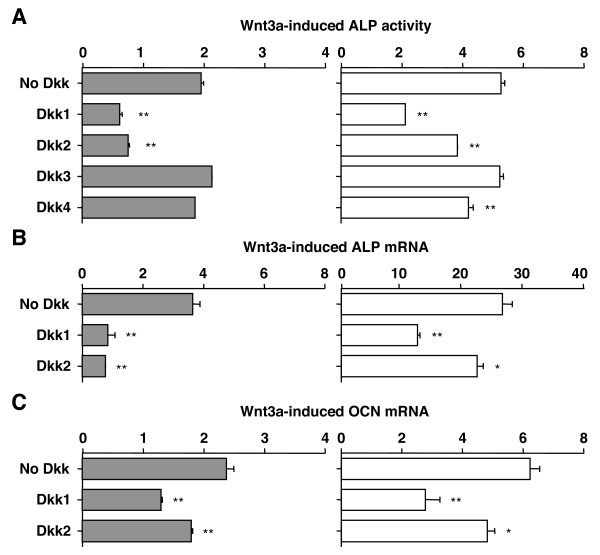
**Dkk1 and -2 inhibit Wnt3a-induced expression of osteoblastic differentiation markers in C2C12 cells (left) and C3H10T1/2 cells (right). ***A*, Inhibition of Wnt3a-induced ALP activity by Dkk1 and -2 in cell lines C2C12 (*grey columns*) and C3H10T1/2 (*white columns*). Cells were pre-incubated for 3 h with Dkk1-4-CM or M-CM. Wnt3a-CM was added and cells were cultured for six days. L-CM was used in control experiments to determine the baseline level of ALP in cells not stimulated with Wnt3a (not shown). ALP activity was measured by ELISA and values were normalized to total protein. Results are presented as fold induction relative to cells not stimulated with Wnt3a. Data are means ± SD from three independent experiments. Asterisks indicate significant decreases (*p *< 0.005) compared to cells not treated with Dkk. *B*, Inhibition of Wnt3a-induced ALP mRNA by Dkk1 and -2. Experimental setup was the same as described in panel A except cells were stimulated with Wnt3a for 3 rather than 6 days. ALP mRNA was determined by real-time RT-PCR and normalized to the message levels of β-actin mRNA. Data are means ± SD from three independent experiments. One asterisk (*p *< 0.05) and two asterisks (*p *< 0.005) denote a significant loss of ALP message compared to cells not treated with Dkk. *C*, Inhibition of Wnt3a-induced OCN mRNA expressions by Dkk1 and -2. Experimental setup was the same as described in panel A. OCN mRNA was determined by real-time RT-PCR and normalized to β-actin message levels. Data are means ± SD from three independent experiments. One asterisk (*p *< 0.05) and two asterisks (*p *< 0.005) denote a significant drop in OCN message relative to cells not treated with Dkk.

To investigate the role of Dkk proteins in Wnt-induced osteoblast differentiation *in vitro*, we took advantage of cell lines C3H10T1/2 and C2C12. The former is a mouse mesenchymal stem cell with differentiation potential along several lineages including osteoprogenitors. The latter is a primitive mouse myoblast that can undergo differentiation to preosteoblast-like cells. In both cell lines, osteoblastic differentiation can be monitored by expression of ALP and OCN, two representatives of early and late markers of osteoblast differentiation, respectively. Treatment with Wnt3a led to a 5-fold increase in ALP activity in C3H10T1/2 cells (Fig. 2A right, top bar), confirming a previous result under similar conditions [10]. The ALP increase in C2C12 cells was not as pronounced (2-fold) but clearly present (Fig. 2A left, top bar).

Treatment of both cell lines with Dkk1 and -2 resulted in a significant drop in Wnt3a-induced ALP (Fig. 2A). Unlike C2C12 cells, treatment of C3H10T1/2 cells with Dkk4 led to a small but highly significant decrease in ALP. This indicated that Dkk4 sometimes exhibited Wnt-inhibiting activity, as mentioned above. Dkk3 was ineffective in both cell lines, as expected (Fig. 2A). The ALP-inhibiting effect of Dkk1 and -2 was confirmed at the level of gene transcription using real-time RT-PCR (Fig. 2B). Just as in case of ALP protein, Dkk1 was a potent inhibitor in both cell lines, whereas Dkk2's inhibitory activity was more convincing in C2C12 than in C3H10T1/2 cells. In contrast to Dkk3 and -4 (results not shown), Dkk1 and -2 inhibited the Wnt3a-dependent induction of OCN mRNA (Fig. 2C), with Dkk2 being once again more potent in C2C12 than C3H10T1/2 cells. These quantitative differences aside, the results suggested that Dkk1 and -2 inhibit Wnt/β-catenin-dependent osteoblast differentiation.

### Dkk1 and -2 enhance BMP2-induced ALP expression

**Figure 3:**
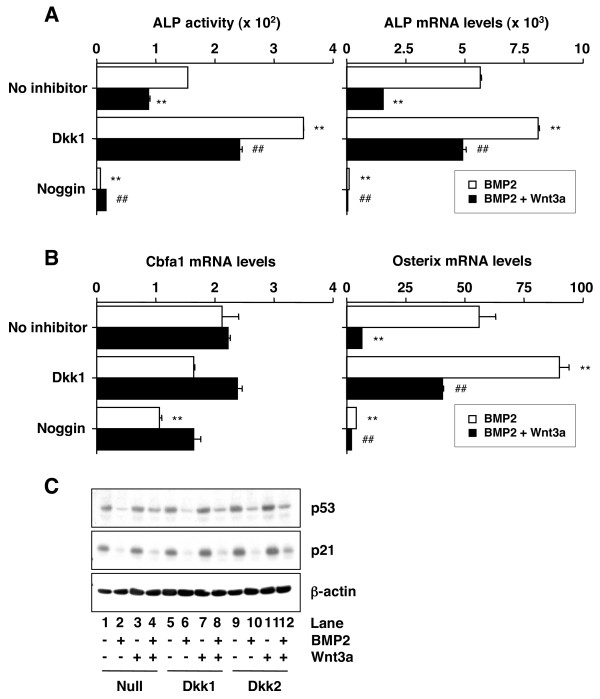
**Wnt3a inhibits ALP and Osterix, but restores p53, in BMP2-stimulated C2C12 cells**. *A*, Wnt3a diminishes BMP2-dependent expression of ALP. Cells were pre-cultured for 3 h with M-CM (not containing Wnt or BMP inhibitor), Dkk1-CM, or M-CM supplemented with 100 ng/ml Noggin as indicated. L-CM (no Wnt3a) supplemented with 100 ng/ml BMP2 (*white columns*) or Wnt3a-CM supplemented with the same amount of BMP2 (*black bar*) was added and cells were cultured for 3 days in case of mRNA measurement or 6 days in case of protein determination. Assays were performed as described in Figure 2. Results are presented as fold induction relative to cells not stimulated with BMP2 or BMP2/Wnt3a. Data are means ± SD from three independent experiments. Asterisks and number signs indicate significant changes (*p *< 0.005) compared to treatment with BMP2 only (top bar) and BMP2 plus Wnt3a (2^nd ^bar from top), respectively. *B*, Wnt3a inhibits BMP2-induced expression of Osterix but leaves Cbfa1 unchanged. Message levels of both genes were determined by real-time RT-PCR. Cells were treated and results are presented as described in panel A. *C*, Wnt3a mitigates the BMP2-dependent loss of p53. Cells were cultured for 3 days as indicated in panel A. Protein levels of p53 (*top*), p21 (*center*) and β-actin (*bottom*) were determined by Western blotting using 20 μg cell lysate per lane. The same result was obtained in two additional experiments (not shown).

The developmental cues emanating from BMP signaling must be integrated with inputs from Wnt and other signal transduction pathways to properly orchestrate the network of transcription factors that govern osteoblast differentiation. Based on this consideration, we assessed osteoblastogenesis under conditions of ongoing BMP or Wnt/BMP signaling further modulated by the BMP inhibitor, Noggin, and the Wnt inhibitor, Dickkopf. Similar to previous reports on BMP2-induced differentiation of preosteoblasts [36,37], treatment of C2C12 cells with BMP2 led to a substantial induction of ALP protein (~150-fold, Fig. 3A left, top bar) and ALP mRNA (~6000-fold, Fig. 3A right, top bar). This response was abrogated by Noggin (2^nd ^bar from bottom), indicating that ALP induction was dependent on BMP signaling. Surprisingly, we also found that in BMP2-stimulated cells, addition of Wnt3a resulted in a significant down-regulation of ALP (Fig. 3A, 2^nd ^bar from top), even though Wnt3a weakly induced ALP in absence of BMP2 (Fig. 2A). Treatment of BMP2-stimulated C2C12 cells with Dkk1 led to a more than two-fold increase in ALP protein (Fig. 3A left, 3^rd ^bar from top), not a decrease as reported elsewhere [27]. The increase was blunted by Wnt3a (Fig. 3A left, 4th bar), indicating that the Dkk1 effect was indeed dependent upon Wnt signaling, not caused by some unrelated property of this inhibitor. Dkk2 shared the above-described activities of Dkk1, while Dkk3 and -4 were ineffective (data not shown). These results indicated that in BMP2-stimulated osteoprogenitors the Wnt3a-dependent inhibition of ALP is partially abrogated by Dkk1 and -2.

### Dkk1 and -2 enhance BMP2-induced Osterix expression

Because transcription factors Cbfa1, Osterix and ATF4 are instrumental for the cell fate decision that commits mesenchymal progenitors to undergo differentiation to osteoblasts [30,31,38], we sought to determine whether Wnt3a/BMP2 signaling influences the expression of these transcription factors in differentiating C2C12 cells. Using real-time RT-PCR, we found that BMP2 caused but a modest (2-fold) increase in Cbfa1 mRNA (Fig. 3B left, top bar), yet a substantial (~60-fold) elevation in Osterix mRNAs (Fig. 3B right, top bar). ATF4 message was unchanged (data not shown). The induction of the Cbfa1 and Osterix genes was inhibited by Noggin (2^nd ^bar from bottom), indicating that the transcriptional activation relied on BMP2 signaling. Similar to ALP, the expression of Osterix, but not Cbfa1, was down regulated by Wnt3a (Fig. 3B right, 2^nd ^bar from top). Treatment of BMP2- or BMP2/Wnt3a-stimulated cells with Dkk1 resulted in both cases in a marked elevation of Osterix message (3^rd ^bar and 4^th ^bar, respectively), further indicating that Wnt signaling is a suppressor of Osterix. Dkk2 shared the above-described activities of Dkk1, while Dkk3 and -4 were ineffective (data not shown). These findings suggested that Wnt/β-catenin inhibits BMP2-dependent induction of Osterix in osteoprogenitors.

### Wnt3a restores BMP2-dependent loss of p53

Osterix expression in osteoblasts has been recently shown to be negatively regulated by p53 [32]. We therefore speculated that the BMP2- or Wnt3a-dependent up- or down-regulation of Osterix may be accompanied by corresponding changes in p53 levels: decrease of p53 in case of BMP2 treatment and increase of p53 in case of treatment with Wnt3a. To evaluate this, we determined p53 in C2C12 cells using Western blotting. Lysate from untreated cells contained levels of p53 that were markedly reduced upon treatment with BMP2 (Fig. 3C, lanes 1 and 2). Treatment of cells with Wnt3a, which left the p53 baseline essentially unaffected (lane 3), restored p53 to near baseline levels in the presence of BMP2 (lane 4). Addition of Dkk1 or -2 did not alter the levels of p53 in Wnt3a-stimulated cells (compare lanes 7 and 11 with lane 3), but slightly attenuated the Wnt3a-dependent restoration of p53 under conditions of ongoing BMP2 signaling (compare lanes 8 and 12 with lane 4). Since p53 is a positive regulator of p21 expression, we next examined whether the BMP2-dependent loss of p53 was associated with the reduction of p21. Figure 3C (center) shows that this was the case. In fact, the changes of p21 closely matched those of p53 regardless of the specific treatment condition. These findings suggested that Wnt/β-catenin signaling by itself is not important for the regulation of p53/p21 in preosteoblasts. However, in cells undergoing BMP2 signaling, the Wnt/β-catenin pathway functions as a strong antagonist of the BMP-mediated suppression of p53.

### Dkk1 and -2 inhibit Wnt3a-induced OPG production

**Figure 4:**
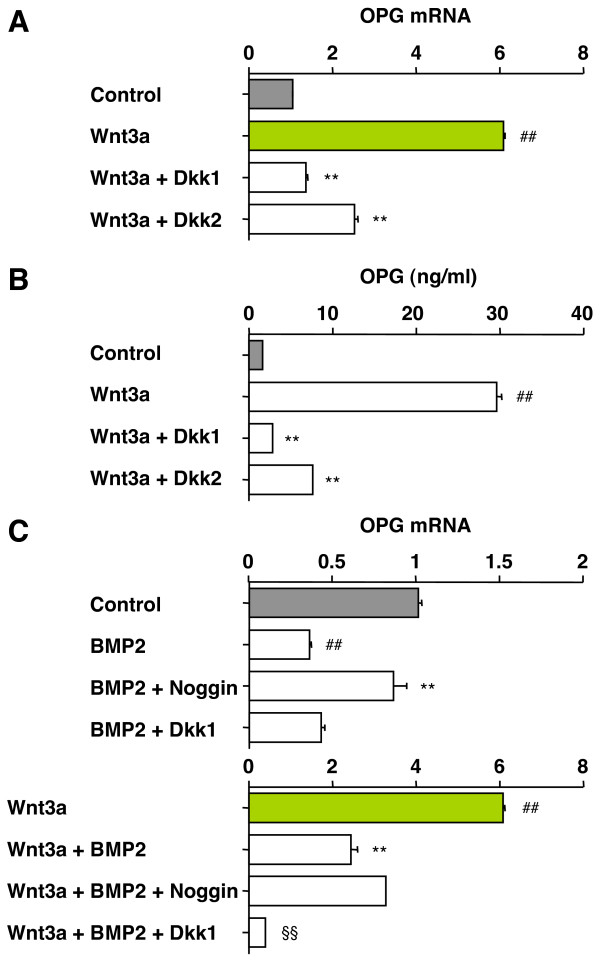
**Dkk1 and -2 inhibit Wnt3a-induced OPG expression in C2C12 cells**. *A*, Inhibition of Wnt3a-induced OPG mRNA by Dkk1 and -2. Cells were pre-cultured in Dkk1- or Dkk2-CM for 3 h as indicated. M-CM (no Dkk) was used for the two samples at the top. Wnt3a-CM or L-CM (no Wnt 3a; control) was added and cells were grown for 3 days. Total RNA was extracted and OPG mRNA was determined using real-time RT-PCR. Message levels were normalized using β-actin mRNA as reference. Results are presented as fold induction relative to the control. Data are shown as means ± SD based on 5 independent experiments. Number signs and asterisks indicate significant changes (*p *< 0.005) compared to cells not treated with Wnt3a (top bar) and treated with Wnt3a (2^nd ^bar from top), respectively. *B*, Dkk1 and -2 dependent inhibition of Wnt3a-induced OPG protein secreted into the extracellular milieu. Cells were treated as described above, supernatant was harvested, and OPG was determined using ELISA. Results are presented as in panel A. *C*, Wnt3a-dependent OPG induction is inhibited by BMP2. Cells were pre-incubated for 3 h with Dkk1-CM or M-CM supplemented with 100 ng/ml Noggin where indicated. Cells were grown for 3 days in L-CM (*top panel*) or Wnt3a-CM (*bottom panel*) that contained 100 ng/ml BMP2 where indicated. OPG mRNA was measured and results were calculated as described in panel A. Number signs, asterisks and paragraph symbols indicate significant changes (*p *< 0.005) compared to untreated cells (control), BMP2-treated cells and BMP2/Wnt3a-treated cells, respectively. Note that the bars labeled Control (black) and Wnt3a (green) in this panel are identical to corresponding bars in panels A and B.

Wnt3a/β-catenin signaling has been reported to induce OPG transcription in C3H10T1/2 cells [13], but this has not yet been extended to C2C12 cells, and the influence of Dkk proteins and ongoing BMP2 signaling on OPG expression are also unknown in both cell types. To that end, we found that treatment of C2C12 cells with Wnt3a resulted in a 6-fold induction of OPG mRNA (Fig. 4A, 2^nd ^bar from top). This induction was abrogated by Dkk1 (3^rd ^bar) and strongly inhibited by Dkk2 (4^th ^bar). Dkk3 and -4 were ineffective (not shown). Similar findings were obtained with C3H10T1/2 cells (not shown). To further validate these results, we used ELISA to measure the amount of OPG protein secreted into the cell culture supernatant. There was a good match of OPG message and protein (Fig. 4B) for all four treatment conditions. The same result was obtained with C3H10T1/2 cells (not shown). We next measured OPG mRNA in BMP2-stimulated and BMP2/Wnt3a-costimulated C2C12 cells. BMP2 decreased OPG mRNA below the level seen in the control (Fig. 4C top, 2^nd ^bar). The decrease was abolished by Noggin (3^rd ^bar), but not by Dkk1 (4^th ^bar). Wnt3a partially restored the level of OPG mRNA held down by treatment of cells with BMP2 (Fig. 4C bottom, 2^nd ^bar). The restoration was abolished by Dkk1 (4^th ^bar), but not by Noggin (3^rd ^bar). These results demonstrated that OPG is regulated by both Wnt/β-catenin and BMP2 signaling. The Wnt pathway induces OPG, whereas the BMP2 pathway suppresses OPG. Interestingly, Dkk1 kept OPG low irrespective of the BMP2 signaling status (on or off). This pointed to a mechanism by which Dkk1 may promote osteolytic lesions *in vivo*: enhancement of RANKL-mediated osteoclastogenesis.

### Wnt3a suppresses basal and 1,25(OH)2D3-induced RANKL, but not BMP2-induced RANKL

**Figure 5:**
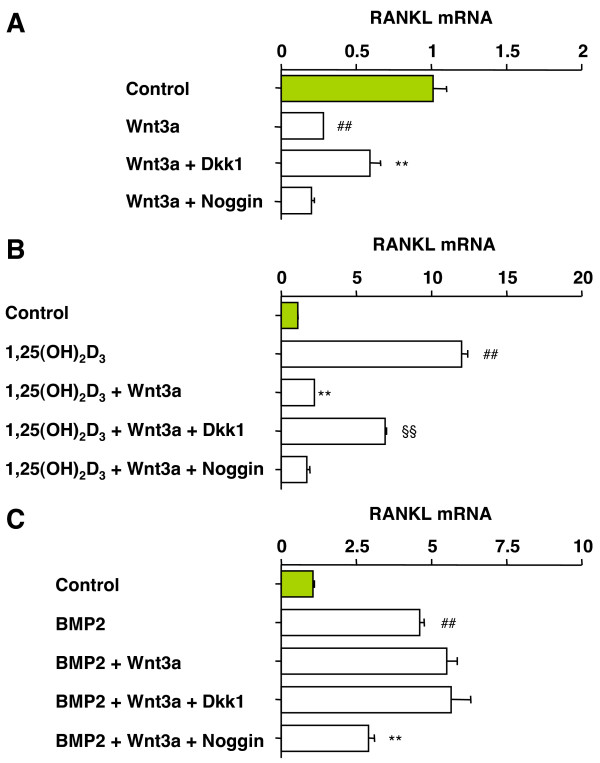
**Dkk1 attenuates Wnt3a-dependent inhibition of RANKL in C2C12 cells treated with 1,25(OH)**_2_**D**_3_**or left untreated**. *A*, Mitigation of Wnt3a-dependent suppression of RANKL message by Dkk1. Cells were pre-incubated for 3 h in Dkk1-CM or M-CM supplemented with 100 ng/ml Noggin. Plain M-CM was used for the two samples at the top (not indicated). Cells were grown for 3 days in Wnt3a-CM or L-CM without Wnt3a (control). Total RNA was extracted and mRNA levels of RANKL were determined using real-time RT-PCR. Data are shown as means ± SD based on 5 independent experiments. Number signs and asterisks designate a significant drop (*p *< 0.005) compared to the control and Wnt3a-treated cells, respectively. *B*, Dkk1 partially restores the Wnt3a-mediated drop of RANKL mRNA in cells treated with 1,25(OH)_2_D_3_. Cells were pre-incubated as desribed in panel A and grown for 3 days in L-CM (no Wnt3a) or Wnt3a-CM supplemented with 10 nM 1,25(OH)_2_D_3_. Data are shown as means ± SD based on 5 independent experiments. Number signs, asterisks and paragraph symbols designate significant changes (*p *< 0.005) compared to untreated cells, 1,25(OH)_2_D_3_-treated cells and Wnt3a/1,25(OH)_2_D_3_-treated cells, respectively. *C*, Wnt3a does not affect BMP2-dependent RANKL expression. Cells were pre-incubated as described in panel A and grown for 3 days in Dkk1-CM (2^nd ^bar from bottom), M-CM supplemented with 100 ng/ml Noggin (bottom bar), or M-CM (3 top bars). Cells were grown for 3 days in L-CM (top bar), L-CM containing 100 ng/ml BMP2 (2^nd ^bar from top), or Wnt3a-CM supplemented with 100 ng/ml BMP2 (three bars at bottom). Data are shown as means ± SD from 3 independent experiments. Number signs and asterisks designate a significant elevation compared to untreated cells and a reduction compared to BMP2-treated cells (*p *< 0.005), respectively. Note that the bar labeled Control (green) in this panel is identical to corresponding bars in panels A and B.

Wnt/β-catenin signaling has been demonstrated to down-regulate RANKL mRNA in MC3T3-E1 preosteoblast [14], but thus far this situation has not been studied in C2C12 or C3H10T1/2 cells. The influence of Dkk proteins on RANKL expression and the outcome of possible crosstalk of the Wnt, BMP2 and 1,25(OH)_2_D_3 _signaling pathways are also unknown. On this backdrop, we found that Wnt3a repressed RANKL mRNA in C2C12 cells (Fig. 5A) and C3H10T1/2 cells (results not shown). In both cell lines RANKL suppression was mitigated by Dkk1, whereas Dkk2, -3 and -4 and Noggin were ineffective (Fig. 5A and data not shown). Wnt3a also repressed 1,25(OH)_2_D_3_-induced RANKL mRNA expression in C2C12 cells (Fig. 5B), which was partially reversed by Dkk1 but not by Noggin (Fig. 5B), indicating involvement of the Wnt/β-catenin pathway. BMP2 increased RANKL mRNA in C2C12 cells (Fig. 5C), which was inhibited by Noggin, as expected. Importantly, because Wnt3a was unable to down regulate the BMP2-dependent induction of RANKL, the addition of Dkk1 to this system was inconsequential (Fig. 5C). This indicated that in contrast to 1,25(OH)_2_D_3_-stimulated and untreated C2C12 cells, cells undergoing BMP signaling become unresponsive to Wnt/β-catenin's inhibiting effects on RANKL expression.

### Dkk1 abrogates the suppressive effect of Wnt3a on M-CSF production in BMP2-stimulated cells

**Figure 6:**
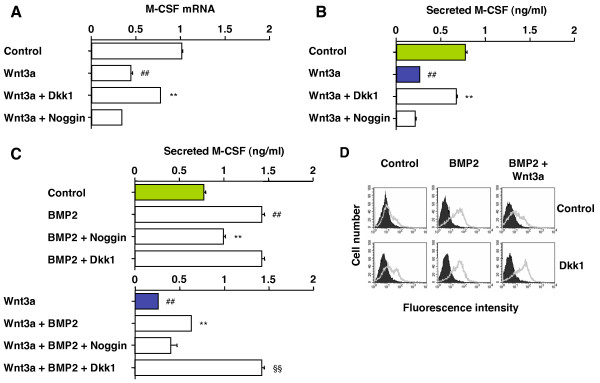
**Dkk1 restores Wnt3a-dependent loss of M-CSF in C2C12 cells**. *A*, Dkk1 restores Wnt3a-dependent decrease in M-CSF message. Cells were pre-incubated for 3 h in Dkk1-CM, M-CM supplemented with 100 ng/ml Noggin, or plain M-CM (two columns at the top). Cells were grown for 3 days in Wnt3a-CM or L-CM that did not contain Wnt3a (control). Total RNA was extracted and mRNA levels of M-CSF were determined using real-time RT-PCR. Data are shown as means ± SD based on 5 independent experiments. Number signs and asterisks indicate significant changes (*p *< 0.005) compared to untreated cells and Wnt3a-treated cells, respectively. *B*, Dkk1 restores Wnt3a-dependent decrease in M-CSF protein secreted into the extracellular milieu. Supernatant was harvested and M-CSF was determined using ELISA. Cells were treated and results are presented as described above. *C*, Wnt3a-dependent loss of secreted M-CSF is abrogated in BMP2-treated cells by Dkk1. Cells were pre-incubated as described in panel A. Cells were grown for 3 days in L-CM (*top panel*) or Wnt3a-CM (*bottom panel*), both containing 100 ng/ml BMP2 where indicated. Cells cultured in L-CM (no Wnt3a) served as control. Supernatant was harvested and M-CSF was determined using ELISA. Results are shown as means ± SD based on 5 independent experiments. Number signs, asterisks and paragraph symbols denote significant differences (*p *< 0.005) compared to untreated cells, BMP2-treated cells and Wnt3a/BMP2-treated cells, respectively. Note that the control here is identical to the control in panel B and that the top-most bar in the bottom panel is identical to the "Wnt3a" bar in panel B. *D*, Wnt3a-dependent loss of membrane-associated M-CSF is abrogated in BMP2-stimulated cells by treatment with Dkk1. Cells were pre-incubated and cultured as in panel C followed by FACS analysis using antibody to M-CSF (open histograms) or isotype-matched unrelated antibody (filled histograms). BMP2-stimulated cells or cells left untreated demonstrated very small, if any, changes in surface M-CSF expression upon treatment with Dkk1. Cells co-stimulated with BMP2 and Wnt3a, however, showed a marked increase in M-CSF reactivity in presence of Dkk1. Similar results were obtained in three independent experiments.

Osteoblast-produced M-CSF, a positive regulator of osteoblast-dependent osteoclast development, might be influenced by Wnt or BMP2 signaling in mesenchymal stem cells undergoing osteoblastic differentiation. To investigate this, we used C2C12 as the model system and real-time PCR as the measurement tool. We found that Wnt3a repressed M-CSF mRNA compared to the control (Fig. 6A, 2^nd ^bar from top). Unlike Noggin, Dkk1 restored the M-CSF message, further indicating that M-CSF is down regulated by Wnt/β-catenin signaling in this cell type. ELISA of C2C12 cell culture supernatant confirmed these findings at the level of secreted, soluble M-CSF protein (Fig. 6B). We next evaluated M-CSF production in C2C12 cells undergoing BMP2 and Wnt3a/BMP2 signaling further modulated by Noggin and Dkk1. BMP2 enhanced M-CSF by a factor of two (Fig. 6C top). Noggin, but not Dkk1, blocked the response. Wnt3a repressed the BMP2-induced increase of M-CSF in a manner that was reversed by Dkk1 but not Noggin (Fig. 6C bottom), confirming that the Wnt/β-catenin pathway is a negative regulator of M-CSF. FACS analysis of the same cell preparation evaluated in panel C demonstrated a good match of the secreted and membrane-associated forms of M-CSF (Fig. 6D). These results demonstrated that M-CSF expression in osteoblast progenitors is up and down regulated by BMP and Wnt/β-catenin signaling, respectively. Dkk1 stabilized M-CSF expression levels in presence and absence of BMP2, suggesting yet another mechanism by which Dkk1 activates osteoclast development *in vivo*.

## Discussion

The present study used mesenchymal progenitor cells undergoing osteoblastic differentiation *in vitro *to demonstrate that Wnt inhibitors Dkk1 and -2 and, to a lesser extent, Dkk4 enhance the osteoclastogenic potency of early osteoprogenitors. The underlying mechanism appears to involve at least two aspects of Dickkopf biology. The first concerns the restoration of the Wnt/β-catenin-induced reduction of M-CSF in BMP2-stimulated osteoprogenitors. The second involves the ability of Dkk1 and -2 to mitigate the Wnt/β-catenin-dependent down-regulation of RANKL (observed in 1,25(OH)_2_D_3_-stimulated osteoprogenitors) and the concomitant up-regulation of its inhibitor, OPG (seen in BMP2-induced osteoprogenitors). We postulate that the resultant changes in M-CSF/c-Fms and RANKL-RANK signaling enhance osteoclast development *in vivo*; i.e., the differentiation of monocytic precursors via pre-osteoclasts to mature, activated osteoclasts. The Dkk1- and -2-dependent changes in M-CSF, RANKL and OPG expression, and the somewhat unexpected ability of Dkk1 and -2 to promote rather than inhibit certain aspects of osteoblastogenesis, will be discussed in greater depth in the following.

Our finding that Dkk1 and -2 abolished the Wnt3a-dependent down regulation of ALP message and protein in otherwise untreated (Fig. 2) or BMP2-stimulated C2C12 and C3H10T1/2 cells (Fig. 3) indicated that Wnt3a inhibits certain aspects of osteoprogenitor differentiation *in vitro*. The mitigating effects of Wnt3a in BMP2-induced cells were consistent with results from other investigators indicating extensive crosstalk of the Wnt/β-catenin and BMP signaling pathways in pre-osteoblasts. Pertinent observations include the activation of Wnt/β-catenin by BMP2 [39]; inhibition of BMP2-induced ALP by Dkk1 [40]; conversely, enhancement of BMP2-induced ALP by enforced expression of constitutively active β-catenin [41]; and inhibition of Wnt3a-dependent ALP by Noggin [42]. Recent work with mouse C2C12 and human mesenchymal stem cells showed that TGF-β1 inhibits BMP2-induced ALP in a manner that involves the translocation of β-catenin to the cell nucleus [43,44]. This has connected TGF-β signaling with the BMP and Wnt/β-catenin pathways. Our findings are also in agreement with the notion that Wnt/β-catenin inhibits BMP2-dependent osteoprogenitor development by a mechanism that relies on the nuclear translocation ofβ-catenin, although this has not yet been formally demonstrated.

**Figure 7:**
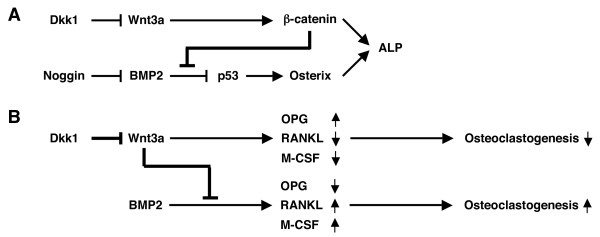
**Hypothetical scheme of the mechanism by which Dkk1 promotes osteolytic lesions *in vivo***. *A*, The cellular differentiation of osteoprogenitors, which can be monitored by Osterix and ALP expression, is governed in part by Wnt3a and BMP2 signaling. BMP2, which is inhibited by Noggin, is a strong inducer of Osterix and ALP, relying on an indirect mechanism that involves p53 and Cbfa1 (not included). Wnt3a/β-catenin signaling, which is antagonized by Dkk1 (and Dkk2), can have opposing effects on osteoblast development depending on BMP2 signaling. In absence of BMP2, Wnt3a weakly promotes osteoblast differentiation through the β-catenin pathway. In presence of BMP2, Wnt3a inhibits osteoblast differentiation by down-regulating the BMP2-p53-Cbaf1-Osterix-ALP axis (thick line). The molecular mechanism of the crosstalk has not yet been elucidated. Our scheme postulates that the Dkk1-dependent inhibition of Wnt3a's positive effect on osteoprogenitors *in vitro *may be outweighed by the abrogation of Wnt3a's negative effect on BMP2 signaling, such that, on balance, Dkk1 enhances features of early osteoblastogenesis. *B*, The differentiation of osteoclasts, which is strictly dependent upon interaction of osteoclast precursors with osteoblasts, is mediated in part by signaling pathways that emanate from the binding of M-CSF and RANKL to c-Fms and RANK, respectively. Osteoblasts stimulated by BMP2 express high levels of M-CSF and RANKL (positive regulators of osteoclastogenesis) but low levels of OPG (negative regulator of osteoclastogenesis), resulting in a positive signal for osteoclast formation. Osteoblasts exposed to Wnt3a exhibit the opposite phenotype with regard to M-CSF, RANKL and OPG expression, resulting in a negative signal for osteoclast formation. Osteoclastogenesis is further dampened by Wnt3a's ability to inhibit BMP2 signaling, as indicated in panel A (thick line). Constitutive expression of Dkk1 (and Dkk2) by bone marrow-homing tumor cells (e.g., multiple myeloma) may enhance osteoclastogenesis *in vivo *by inhibiting Wnt3a, thus shifting the balance of Wnt3a/BMP2 co-signaling in favor of BMP2 signaling. The consequent increase in osteoclastogenesis promotes lytic bone lesions as long as the paracrine supply of Dkk1 by tumor (myeloma) cells is sustained.

Our observation that Wnt3a blocked the BMP2-induced expression of Osterix [30] raised questions about the mechanism by which Wnt/β-catenin suppresses BMP-dependent osteoblast differentiation. We showed, for the first time, that the expression of p53, a negative regulator of Osterix [32], is down regulated by BMP2. Furthermore, Wnt3a was able to restore the BMP2-dependent loss of p53. These results supported the model that BMP2-dependent osteoblast differentiation is controlled in large measure by the p53-Cbfa1-Osterix-ALP axis (Fig. 7A). Apparently, this axis is down modulated by crosstalk with the Wnt/β-catenin pathway, the precise molecular mechanism of which remains to be elucidated. An important implication of our hypothetical scheme is that Dkk1 and -2 may promote osteoblast differentiation by virtue of releasing the β-catenin-dependent brake on the BMP-p53-Cbfa1-Osterix-ALP pathway.

Expression of OPG in osteoblasts, a crucial factor for maintaining normal bone mass, has been reported to be regulated by Wnt/β-catenin signaling [11-13]. We confirmed this in three different types of osteoprogenitors (results for MC3T3-E1 cells not shown); demonstrated for the first time that Dkk1 and -2 inhibit the Wnt3a-induced OPG expression at the mRNA and protein level; and further showed that the BMP2-dependent repression of OPG is not affected by Dkk1 or -2 (Fig. 4). Because osteoblast development *in vivo *is likely to occur in the co-presence of Wnt and BMP ligands, it seemed important to evaluate OPG expression upon co-stimulation of cells with Wnt3a and BMP2. Our results indicated that Wnt signaling predominates over BMP signaling in this regard. The increase in OPG output in Wnt3a-treated cells was abolished by Dkk1 and -2, suggesting that abnormally high levels of Dkk1 or -2 *in situ *reduce the amount of OPG secreted into the extracellular milieu. The resultant promotion of RANKL-dependent osteoclastogenesis may contribute to the development of lytic bone lesions in MM [27]. In support of this theory, MM patients generally exhibit reduced OPG serum levels compared to healthy individuals, and the number of lytic bone lesions is inversely correlated with OPG [45,46].

Wnt/β-catenin signaling has been reported to attenuate osteoclastogenesis by down modulating the important regulator of bone homeostasis, RANKL [47], in osteoprogenitors [14]. We extended this observation to the present C2C12 and C3H10T1/2 cells and the 1,25(OH)_2_D_3_-induced pathway of RANKL activation in these cells (Fig. 5). We further showed, for the first time, that M-CSF was down regulated by Wnt/β-catenin in BMP2-stimulated osteoprogenitors (Fig. 6). M-CSF is a positive regulator of Osterix, which, in turn, down regulates p53 [32]. Our observation that BMP2-regulated p53 levels were inversely correlated with levels of Osterix and M-CSF suggested that, just like ALP, M-CSF is controlled by the BMP-p53-Osterix axis. Additional studies are warranted to validate this hypothesis. Studying the impact of Wnt3a on BMP2- or 1,25(OH)_2_D_3_-dependent expression of RANKL, we noted that Wnt3a markedly diminished the 1,25(OH)_2_D_3_-induced RANKL but did not interfere with the BMP2-induced RANKL. Thus, among different pathways of RANKL induction, only a subset appears to be prone to down modulation by Wnt/β-catenin. Dkk1 and -2 partially restored the Wnt3a-dependent reduction of RANKL in cells treated with 1,25(OH)_2_D_3_, pointing to another molecular mechanism by which canonical Wnt inhibitors may promote osteoclastogenesis *in situ*.

This study systematically compared Dkk1 with the three other members of the Dickkopf family. Although only selected data are presented in this report, all experiments involving Dkk1 also included Dkk2, -3 and -4. In agreement with other studies [33,48], Dkk3 lacked activity in any of the Wnt3a-mediated responses evaluated here. This was different for Dkk2 and -4, which recapitulated some features of Dkk1. Dkk2 tended to be more active in C2C12 than C3H10T1/2 cells, whereas the opposite was true for Dkk4. Considering the activity of Dkk2 and -4, it is interesting to note that only Dkk1 has been implicated thus far in cancer-associated bone lesions. Unlike Dkk2 and -4, Dkk1 binds to Kremen1/2, which leads to rapid endocytosis of the Dkk1-LRP5/6-Kremen1/2 complex [49]. This raises the possibility that Kremen is a critical co-factor of Dkk1-dependent osteolysis that warrants further investigation.

## Conclusion

The present study further implicates impaired Wnt signaling in the pathogenesis of cancer-associated lytic bone lesions. Our results support the hypothesis that the constitutive secretion of Dickkopf by tumor cells leads to focal bone loss by a complex mechanism that involves enhanced osteoclastogenesis (Fig. 7B). *In vivo *approaches will be necessary to further test this hypothesis. To that end, the Dkk1-4-secreting sublines of the mouse plasmacytoma MOPC315 developed here may provide a starting point, as the tumor cells can be readily transplanted into BALB/c mice where they infiltrate the bone marrow (results not shown) and, possibly, cause osteolytic lesions in dependence on Dickkopf expression. Pre-clinical studies of this sort may also facilitate the development of small-drug Dkk inhibitors that may be useful not only for the treatment and prevention of cancer-associated bone loss, but also for treatment of osteoporosis, periodontal disease and bone destruction consequent to rheumatoid arthritis. Success will require further assessment of the molecular properties of the various Dkk's, their specific activities in different target cells, and thorough understanding of the regulatory loops between signaling pathways and cell populations affected by Wnt signaling in bone.

## Methods

### Cell lines and cell culture

Mouse plasmacytoma MOPC315.4 [50], a generous gift from Dr. B. Bogen (University of Oslo, Oslo, Norway), was cultured in RPMI 1640 containing 10% fetal bovine serum (FBS), 2 mM glutamine, 50 μM 2-mercaptoethanol, and antibiotics (100 U/ml penicillin, 100 μg/ml streptomycin). Plat-E cells [51] were kindly provided by Dr. T. Kitamura (Institute of Medical Science, University of Tokyo, Tokyo, Japan) and maintained in Dulbecco's modified Eagle's medium (DMEM) containing 10% FBS, 2 mM glutamine, 10 μg/ml blasticidin, 1 μg/ml puromycin, and antibiotics. C2C12 and C3H10T1/2 are mesenchymal stem cells obtained from ATCC (Manassas, VA). Wnt3a-expressing L cells (L-Wnt3a) and the corresponding parental cell line, L, were also purchased from ATCC. Cells were maintained in α-minimal essential medium in the case of C3H10T1/2 cells or DMEM in the case of C2C12, L-Wnt3a and L cells. Both media were supplemented with 10% FBS, 2 mM glutamine, and antibiotics. All cell culture media and supplements were obtained from Invitrogen (Carlsbad, CA). All cells were cultured in a humidified atmosphere at 37°C and 5% CO_2_.

### MOPC315.4 producing Dkk

The cDNAs encoding Dkk1 and -4 were cloned by PCR, using a mouse embryonic cDNA library (Open Biosystems, Huntsville, AL) as template. Dkk2 and -3 cDNAs were obtained by reverse transcriptase-PCR, using total RNA from mouse uterus and brain, respectively, as template. All four cDNAs were modified by adding C-terminal FLAG or His_6 _epitope tags, sequenced, and subcloned into the retroviral expression vector pMY-IRES-EGFP [52] kindly provided by Dr. T. Kitamura (Institute of Medical Science, University of Tokyo). pMY-IRES-EGFP without Dkk insert was used as control. Retrovirus was produced by transient transfection of Plat-E cells. Briefly, 1.6 × 10^6 ^cells were plated in DMEM in 60-mm cell culture dishes for 30 h prior to transfection with retrovirus. Using Lipofectamine 2000 (Invitrogen), cells were transfected with 8 μg of one of the four Dkk expression vectors or the "empty" GFP control vector. The viral supernatant was harvested 16 h later and filter-sterilized. For retroviral infection, 5 × 10^5 ^MOPC315.4 cells were cultured for 16 h in a 6-well plate that contained equal volumes of cell culture medium and retroviral supernatant supplemented with 10 μg/ml polybrene (Sigma, St. Louis, MO). Cells were washed and cultured for 3 days, followed by isolation of virally infected, green fluorescent protein (GFP) expressing cells using a FACSCalibur and the CellQuest software (Becton Dickinson, Franklin Lakes, NJ).

### Conditioned media containing Dkk and Wnt3a

Medium conditioned with Dkk1, -2, -3 or -4 (hereafter referred to Dkk-CM) was obtained from flow-sorted GFP^+ ^plasmacytoma cells. Briefly, 7.5 × 10^5 ^cells were seeded in 75 cm^2 ^flasks containing 25 ml RPMI 1640 supplemented with 10% FBS. After 3 days in culture, supernatant was harvested, filter-sterilized, and stored at 4°C. Medium conditioned with Wnt3a (Wnt3a-CM) was prepared from commercially available L-Wnt3a cells [34]; 1 × 10^6 ^cells were seeded in 100-mm plates containing 10 ml DMEM supplemented with 10% FBS. After cell culture for 4 days, the supernatant was harvested, sterilized, and stored at 4°C. Medium conditioned with flow-sorted GFP^+^Dkk^- ^plasmacytoma cells (M-CM) and parental L cells (L-CM) were used as controls for experiments with Dkk1-4 and Wnt3a, respectively.

### Western Blotting

Cells were incubated on ice for 20 min in CelLytic-MT (Sigma) supplemented with a proteinase inhibitor cocktail (Roche, Indianapolis, IN) followed by centrifugation at 12,000 × *g *for 20 min to prepare whole cell lysate. Protein concentration was determined with the help of the Bio-Rad Protein assay using BSA as standard (Bio-Rad Laboratories, Hercules, CA). Protein (10 μg lysate or 10 μl CM) was incubated for 10 min at 70°C in sample buffer containing reducing reagents; fractionated by SDS-PAGE using 4–12% NuPAGE gels in MES buffer (Invitrogen); transferred to a polivinylidene fluoride membrane (Invitrogen); blocked with StartingBlock T20 buffer (Pierce, Rockford, IL); labeled with un-conjugated primary antibody followed by horseradish peroxidase-conjugated secondary antibody; and visualized with the enhanced chemiluminescence detection system (GE Health Sciences, Piscataway, NJ). The following antibodies/-sera were used: mouse monoclonal to β-actin or FLAG (Sigma), GFP (MBL, Woburn, MA), His_6 _(Invitrogen), or p21 (BD PharMingen); rat monoclonal to Dkk1 or -3 and goat anti-Dkk2 or -4 (R&D Systems, Minneapolis, MN); rabbit anti-p53 (Vector Laboratories, Burlingame, CA).

### β-catenin stabilization

Wnt3a-dependent stabilization of β-catenin was determined as described elsewhere [35]. L cells (4 × 10^5^) were seeded in a 6-well plate, incubated for 24 h, washed, and transferred to 1 ml Dkk-CM or M-CM (control). Following incubation for 3 h, Wnt3a-CM or L-CM was added for 3 h. Cells were lysed in 50 μl lysis buffer. β-catenin was determined by immunoblotting using 10 μg protein as sample and mouse monoclonal anti-β-catenin from BD transduction Laboratories as detection tool. β-catenin levels were quantified by densitometry using NIH Image software. β-catenin expression was normalized by comparison to the housekeeping protein, β-actin.

### Osteoblast differentiation

Cells were seeded at 2 × 10^4 ^cm^-2 ^one day prior to differentiation induction. Cell culture medium was replaced with α-minimal essential medium supplemented with 50 μg/ml ascorbic acid and Dkk-CM, BMP inhibitor (100 ng/ml mouse Noggin [R&D Systems]) or no inhibitor (M-CM). Following pre-incubation for 3 h, cell differentiation was induced by adding Wnt3a-CM and/or L-CM that either contained 100 ng/ml recombinant human BMP2 (R&D Systems) or 10 nM 1,25-dihydroxyvitamine D_3 _(1,25(OH)_2_D_3_, Sigma). Cells were cultured for 3 days (if longer, the differentiation-inducing medium was replaced after 3 days). In some experiments, differentiation occurred in presence of Dkk-CM and/or 100 ng/ml Noggin. Dkk-CM and Wnt3a-CM were added to a final concentration of 20% (v/v), which had been found in pilot studies to result in optimal assay performance in our hands (results not shown).

*Alkaline Phosphatase (ALP)*–ALP activity was determined in cells that had been cultured in 24-well plates for 7 days. Cells were washed twice with phosphate-buffered saline, scraped off the plastic surface with a rubber policeman, and lysed for 15 min in 100 μl Passive Lysis Buffer (Promega, Madison, WI). Cell lysate was spun down at 12,000 × g for 5 min and enzyme activity was determined at 37°C with the ALP LabAssay (Wako Pure Chemical, Tokyo, Japan). Enzyme activity was normalized using total protein (Bio-Rad assay) as reference.

### Real-time RT-PCR

Total RNA was isolated using the RNeasy mini kit (Qiagen, Valencia, CA) supplemented with DNase. First-strand cDNA synthesis was performed using the Superscript III kit (Invitrogen) with 1 μg RNA as template. Real-time PCR was carried out with the ABI 7500 cycler (Applied Biosystems, Foster City, CA). Briefly, 12.5 μl SYBR green PCR master mix (Applied Biosystems) and 5 μl cDNA (five- to ten-fold dilutions of original sample) were added to 200 nM forward and reverse PCR primers in 96-well microplates. Following thorough heat denaturation for 10 min at 95°C, cDNA was amplified in 40 two-step cycles using the following temperature protocol: 95°C for 15 s and 60°C for 60 s. PCR primer sequences and length of amplicons are listed in Table 1. PCR primers were designed by Roche Applied Science as part of their Universal ProbeLibrary. All PCR reactions were performed in triplicates. Results were normalized using β-actin message as reference.

**Table 1:** Primer sequences used for real-time PCR

Target mRNA	Forward primer (5'→ 3')	Reverse primer (5'→ 3')	Product size (bp)
β-actin	TGACAGGATGCAGAAGGAGA	CGCTCAGGAGGAGCAATG	75
ALP	AAGGCTTCTTCTTGCTGGTG	GCCTTACCCTCATGATGTCC	61
ATF4	ATGATGGCTTGGCCAGTG	TCTCCAACATCCAATCTGTCC	72
Cbfa1	GATGACACTGCCACCTCTGA	GCCCAGTTCTGAAGCACCT	81
M-CSF	CAGCTGCTTCACCAAGGACT	TCATGGAAAGTTCGGACACA	60
OCN	AGACTCCGGCGCTACCTT	CTCGTCACAAGCAGGGTTAAG	93
OPG	ATGAACAAGTGGCTGTGCTG	CAGTTTCTGGGTCATAATGCAA	106
Osterix	TTTAAACAAACACGATGATGATGA	ATTGGACTTCCCCCTTCTTG	138
RANKL	TGAAGACACACTACCTGACTCCTG	CCACAATGTGTTGCAGTTCC	83

### Enzyme-linked immunosorbent assay (ELISA)

Cell culture supernatant was harvested 3 days after initiation of osteoblast differentiation as described above. OPG, RANKL and M-CSF were determined by sandwich ELISA, using the respective Duo-Sets from R&D Systems.

### Flow cytometry of M-CSF expression

Cells (1 × 10^6^) were incubated with 0.25 μg biotinylated monoclonal rat antibody to mouse CSF-1 (BD PharMingen) for 1 h at 4°C, followed by incubation with phycoerythin-labeled avidin (eBioscience, San Diego, CA). After washing in phosphate-buffered saline, cells were analyzed on a FACSCalibur using the CellQuest software for data evaluation. Biotinylated rat IgG2a (eBioscience) was used as control for unspecific antibody binding.

### Statistical analysis

Mean values and standard deviations (SD) were calculated and compared using ANOVA followed by Dunnett's multiple comparison *t*-test. *p *values less than 0.05 were considered significant.

## Abbreviations

ALP, alkaline phosphatase

BMP, bone morphogenic protein

CM, conditioned medium

Dkk, Dickkopf

GFP, green fluorescent protein

LRP, low-density-lipoprotein receptor-related protein

M-CSF, macrophage-colony stimulating factor

OPG, osteoprotegerin

RANKL, receptor activator of NF-κB ligand

sFrp, secreted frizzled-related proteins

TGF-β, transforming growth factor-β

1,25(OH)_2_D_3_, 1,25-dihydroxyvitamine D_3_

## Competing interests

The author(s) declare that they have no competing interests.

## Authors' contributions

Ken-ichi Fujita performed all experiments contributed to the design of the study. Siegfried Janz designed the study and wrote and approved the article.
